# Evaluation of China’s High-Speed Rail Station Development and Nearby Human Activity Based on Nighttime Light Images

**DOI:** 10.3390/ijerph18020557

**Published:** 2021-01-11

**Authors:** Zhenyu Du, Wei Wu, Yongxue Liu, Weifeng Zhi, Wanyun Lu

**Affiliations:** 1 School of Geography and Ocean Science, Nanjing University, Nanjing 210023, China; duzhenyu@smail.nju.edu.cn (Z.D.); yongxue@nju.edu.cn (Y.L.); weifengzhi@smail.nju.edu.cn (W.Z.); wanyunlu@smail.nju.edu.cn (W.L.); 2 Key Laboratory of Coastal Zone Exploitation and Protection, Ministry of Natural Resources, Nanjing 210023, China

**Keywords:** high-speed rail (HSR), NPP/VIIRS, brightness, station development, human activity

## Abstract

High-speed rail (HSR) represents China’s advancing productivity; however, quite a few HSR stations face problems due to inappropriate planning and limited passenger flow. To optimize future planning on HSR lines and stations and facilitate efficient operation, we used brightness as a representative of station development and nearby human activity, analyzing its spatial and temporal distribution, classification categories, and influencing factors of 980 stations using nighttime light images from 2012 to 2019. The following conclusions were drawn: (1) There are 41 stations with high brightness between 80 and 320 nW·cm^−2^·sr^−1^, which are concentrated in provincial capitals, large cities, and at line ends. The overall number of these stations increases by 57% in the past eight years. (2) Stations with high brightness but minimal changes that opened in 2013–2019 are mainly concentrated in provincial capitals and large- or medium-sized cities, and those with high brightness and significant changes are mostly new stations nearby. More than 70% of stations that started HSR operation before or in 2012 have high brightness. (3) Brightness positively correlates with the number of daily trains, and it changes faster at stations with more daily trains. It changes most within 0–1 year after HSR operation opening and exhibits a relatively slow but long-term increase over the next 2–6 years.

## 1. Introduction

The construction of transportation infrastructure is a key factor in economic growth [1,2,3,4], which follows a popular maxim in China regarding regional economic development: “If you want to be rich, build roads first” [5]. As a popular transportation mode, the railway is a national economic artery that comprises the backbone of the transportation system, which plays an essential role in China’s economic and social development [6]. Urbanization and economic growth have accelerated since the reform and opening in 1978; the traditional railway network has been unable to meet the growing needs of intercity transportation. Therefore, in January 2004, the State Council approved the “Medium and Long-term Railway Network Plan” proposed by the National Railway Administration, in which the concept of high-speed rail (HSR) was first put forward. The Beijing–Tianjin intercity HSR starting operation on 8 January 2008 is China’s first HSR with independent intellectual property rights, and HSR in China has proliferated since then [7]. The operating HSR mileage reached 35,000 km by 2019, exceeding two-thirds of the total HSR mileage globally.

As of August 2019, 76 lines and 980 stations in China have begun HSR operation. From a macro perspective, four vertical and four horizontal lines comprise China’s HSR network’s primary framework, and eight vertical and eight horizontal lines are expected to be completed by 2030. Most HSR lines are distributed in the southeast of the Hu Huanyong Line, representing uneven geographical development. Only Jining–Baotou, Hohhot–Ordos, and Lanzhou–Urumqi HSR are in the northwestern part. From a micro perspective, the HSR economy has gradually become a brand-new economic form as a typical representative of China’s advanced growth, and many governments have planned to build new HSR towns around the stations. Unfortunately, many HSR station areas have encountered planning issues and small passenger flow, resulting in so-called ghost towns [8], despite high expectations from local governments. There are some successful cases, such as Shanghai Hongqiao Station, Shenzhen North Station, Yuhang Station, and Huizhou South Station. These phenomena indicate an imbalance between the east and west in the layout of HSR lines, and it remains to be verified whether HSR stations can bring enough passenger flow to promote economic development in the surrounding region.

Scholars began to study the impact of HSR on the economy as early as the 1960s [9]. They primarily employ a macro perspective, using socioeconomic statistics to evaluate the impact of HSR on regional and urban economies [9,10,11]. Meng used county-level panel data from 2006 to 2014 in China and found that the HSR network contributes approximately 14% to regional GDP [12]. Ahlfeldt found the HSR linking Cologne and Frankfurt had an average GDP growth of 8.5% [13]. Coto-Millán used cost–benefit analyses and found the introduction of HSR in Spain was not economically justified in 1992 for the Madrid–Seville route, but it is cost-efficient now for the Madrid–Barcelona–French frontier route [14]. Chen found that HSR in Spain had positive effects on the provincial economy growth by stimulating GDP and increasing employment level [15]. It is generally believed that HSR operations have agglomeration and diffusion effects on urban economic activity [16,17]. The diffusion effect is observed in developed areas driving underdeveloped areas, indicating a positive overlap effect, while the agglomeration effect is observed in the flow of production factors from underdeveloped to developed areas, thus inhibiting the development of underdeveloped areas [5]. Many studies concentrate on the factors leading to the agglomeration effect, such as station location, labor migration, and tourism. The location where HSR stations are accessible to the rest of the transport network and having good integration can reinforce the HSR impacts on the local economy [18]. In addition, Guirao et al. used multivariate panel data and found HSR existence allows daily labor migration between cities with HSR stations (separated between 70 and 200 km) and this effect can support agglomeration effects [19,20]. Furthermore, tourist attraction is also an important factors since the HSR boosts tourism by 1.3% more tourists and 1.7% more revenues in Spain [21] and 20.1% and 25.0% respectively in China [22], which in turn leads to agglomeration of population and economy activities.

Moreover, some literature has investigated the balance of HSR and its impact mechanism. The agglomeration and spillover differences in different cities and towns will lead to the imbalance of economic development, resulting in the Matthew effect [10,23,24,25]. Ureña and Vickerman’s studies have shown that large- and medium-sized cities will benefit more than small cities from HSR operation [26,27]. Other studies analyzed the impact mechanism of HSR on economic development and spatial structure. It is concluded that HSR influences regional and urban development by affecting accessibility and production factor flow, which are typically analyzed from an empirical perspective [28]. However, few specific analyses of station development and human activity around HSR stations from a micro perspective, and existing studies on this topic primarily conducted qualitative research of HSR stations [29,30]. Furthermore, socioeconomic statistics exhibit limitations such as lagging, poor scalability, and subjectivity [31]. Remote sensing methods may address the problems mentioned above, providing objective and timely data around HSR stations with a resolution of less than 1 km [32,33].

The remote sensing method is widely used in urban mapping and urban development status analysis [34] because of its advantages, such as comprehensive coverage, high efficiency, and objectivity [35]. Night time light (NTL) uses sensors to record nighttime lights on the surface [36,37], which is a crucial approximation of economic and social development and human activity [38]. Among them, the Defense Meteorological Satellite Program’s Operational Linescan System (DMSP/OLS) data have been widely used since the 1990s [39]. In 2011, Suomi-NPP Visible Infrared Imaging Radiometer Suite (NPP/VIIRS) was used to obtain a new generation of nighttime light data [40]. Compared with DMSP/OLS, it has dramatically improved the spatial resolution and the minimum detection light threshold and solved data saturation issues and lack of on-board calibration [41,42].

The predecessors used nighttime light data as a substitute for social and economic statistics, discussing HSR stations’ impacts on surrounding economic development and its mechanism [43]. The differences-in-differences model is used to simulate and compare scenarios with or without HSR [44]. Zhang Jun et al. selected the counties that opened HSR for the first time in 2008–2013 as the experimental group and used nighttime light data as a proxy variable for the local economic development level. He found that the opening of HSR contributed 34.64% to the economic growth of county-level cities, but the impact of growth is not apparent in counties [5]. Deng et al. used nighttime light data in 2013 to study the development of 124 HSR stations in China within a 2 km radiation area and used multiple linear regression methods to explore the causal mechanisms underlying different development patterns around HSR stations [45]. Zheng et al. used nighttime light data from 2004 to 2013 to study the spatial spillover effect of HSR stations in 97 cities in China and found that the nighttime brightness increased by 27% on average within 4 km of HSR stations [8]. Long et al. used nighttime light data from 2004 to 2013 and found that HSR has a positive impact of 0.12–0.13 on urban expansion, whose role in the central and western regions is almost twice that of the eastern region [16]. Liang et al. used NPP/VIIRS nighttime light data from 2012 to 2017 to quantitatively study the impact of the Guizhou–Guangxi–Guangdong HSR on economic development and found that its influencing factors mainly concentrated on investment effects and industrial structure [46]. These studies only research some HSR lines or stations with DMSP/OLS data; thus, HSR stations in China remain to be comprehensively and more accurately evaluated. In addition, HSR operation-related parameters such as daily trains and operation start time are not included in the study as influencing factors. Furthermore, the level of station development itself and human activity should be discussed. Station development level directly reflects economic and social development; in addition, passenger flow determines the flow of talent, capital, technology, and information to a large extent and therefore determines economic development. Therefore, NPP/VIIRS data of better quality combined with HSR operation-related information should be utilized nationwide to estimate station development and nearby human activity. 

This study used the long time series of NPP/VIIRS data from 2012 to 2019 to analyze the spatial and temporal distribution of the brightness of 980 stations with HSR operation across the country. Then, the stations were classified based on the brightness during HSR operation and relative brightness changes. Finally, the relationship between brightness and HSR daily trains and operation start time was analyzed to determine the impact of HSR operation on brightness. HSR station development and nearby human activity were evaluated to provide a theoretical basis for future HSR line planning, station selection, and operation schedule arrangement to improve the efficiency of HSR and convenience for residents.

## 2. Materials

### 2.1. Study Area

**Figure 1:**
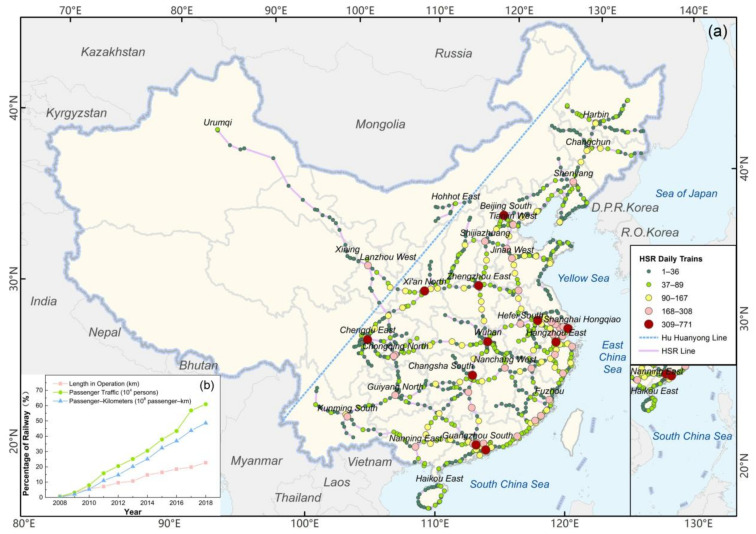
Study area and high-speed rail (HSR) development. (**a**) Study area with HSR lines, stations, and daily trains in 2019. (**b**) HSR development from 2008 to 2018.

China’s HSR is a passenger dedicated line with speeds typically exceeding 250 km/h; in the southwest and southeast mountainous areas, the maximum speed is limited to 200 km/h for safety reasons. Therefore, HSR in this study refers to passenger dedicated lines with speeds greater than 200 km/h [47]. Mainland China was selected as the study area (not including Hong Kong, Macau, and Taiwan), as shown in Figure 1a. Among the 31 provinces, autonomous regions, and municipalities directly under the Central Government, Tibet Autonomous Region, and Ningxia Hui Autonomous Region have no HSR service. As of August 2019, China opened 76 HSR lines, covering 980 stations, regarded as our research objects.

China HSR has been in operation for 12 years from the Beijing–Tianjin intercity railway opening in 2008–2019. At the end of 2018, the operating mileage of HSR increased from 672 to 29,904 km, accounting for more than 20% of railways, and the passenger volume increased from 7.34 million to 2054.3 million people, accounting for more than 60% of railways. Passenger turnover refers to an indicator reflecting the total amount of passenger transportation work within a certain period and is calculated by multiplying the number of passengers transported and the distance traveled [48]. HSR passenger turnover increased from 1.56 billion person-km in 2008 to 687.19 billion person-km in 2018, accounting for almost half of all railway transportation [49], as shown in Figure 1b. HSR has developed rapidly in the past 12 years, bringing great convenience to Chinese travelers.

In terms of station operation status, Guangzhou South Railway Station has the largest scale of services, with 771 HSR daily trains. In addition, there are another 11 stations with daily trains exceeding 300, including Nanjing South, Shanghai Hongqiao, Hangzhou East, Shenzhen North, Changsha South, Beijing South, Zhengzhou East, Wuhan, Chengdu East, Xi’an North, and Jinan West railway stations. These stations are located in the Beijing–Tianjin–Hebei, Yangtze River Delta, Pearl River Delta, and developed provincial capitals in Central and Western China. One-third of the stations have daily trains of less than or equal to 20, and another one-third of stations have daily trains between 21 and 50. Each station’s daily trains are quite different, and many HSR stations in the Northeast, Southwest, and Northwest regions still have great operational potential.

### 2.2. Data Set

The data used in this article mainly include construction and operation data of China HSR, NPP/VIIRS nighttime light monthly composite products, administrative boundaries, and other auxiliary data.

#### 2.2.1. Construction and Operation Data of China HSR

This study used the location data of HSR stations and lines, daily trains, and operation start time of the stations. The HSR stations, lines, and operation start times were taken from the National Railway Administration [50]. Data of daily trains were inquired from the official website of 12306 [51]. Here, we use the train operation chart from 4 November 2019.

#### 2.2.2. Monthly Nighttime Light Data

NPP/VIIRS monthly products were obtained from the Earth Observation Group, National Oceanic, and Atmospheric Administration’s National Geophysical Data Center (NOAA/NGDC). The data remove the effects of scattered light, lightning, lunar illumination, and cloud coverage. The spatial resolution is 15 arc seconds (approximately 450 m at the equator). We downloaded the NPP/VIIRS data from February 2012 to August 2019 within the 20 km buffer zone of the HSR stations from the Google Earth Engine. The average cloud-free observation value in eight years of 980 HSR stations reached 662, which is highly credible (Figure S1).

## 3. Methods

**Figure 2:**
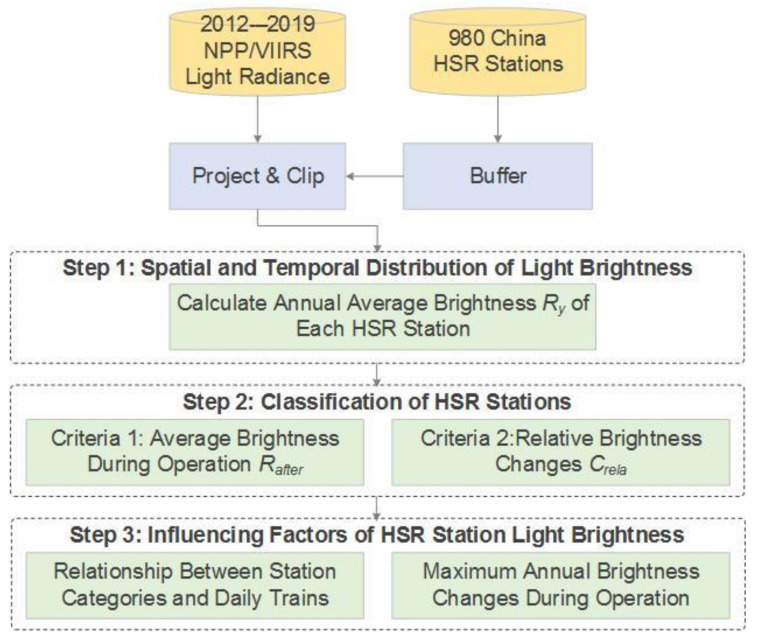
Method flow chart.

Our research methodology was divided into three steps: data preprocessing, classification of HSR stations, and analysis of factors affecting brightness. The flow chart of methods is shown in Figure 2.

### 3.1. Data Preprocessing

The Albers Equal Area Conic Projection was used to ensure that each pixel covers the same area surrounding each target station, and the resolution was unified to 500 m. The brightness of HSR stations was selected to characterize the station development and nearby human activity intensity. We measured several major HSR stations in China, such as Beijing South, Shanghai Hongqiao, Guangzhou South, Shenzhen North, Nanjing South, and Hangzhou East Railway Station, and the size of the stations ranges from 0.5 km × 1.5 km to 1 km × 1.5 km. To eliminate the environmental interference around the stations, we decided to use the maximum brightness within 1.5 km × 1.5 km around the stations to represent the HSR stations’ brightness. After projection and clipping of the original data, we considered 9 pixels for each station in a 3 pixel × 3 pixel area. To select high-quality data, we chose the cloud-free observation value five corresponding to the peak in the frequency distribution histogram as the threshold (Figure S2).

The annual average brightness of each station was calculated according to the monthly light products to increase the results’ reliability because the light at each station varied minimally among months in a year, and the monthly data are easily affected by factors such as seasonal changes and passenger flow. The number of cloud-free observations of each pixel was considered for the weighted average when calculating the station’s annual average brightness, as shown in Formula (1). Among them, *R_y_* is the average value of the maximum brightness within a range of 1.5 km × 1.5 km at a given station in a given year, *R_m_* is the maximum brightness of a given station in a given month, and *C_m_* is the number of cloud-free observations in the month corresponding to the pixel where the maximum brightness is located. Only pixels whose cloud-free observation value is equal to or greater than five were considered.

(1) $$R_{y}=\frac{\sum_{m=1}^{12}\left(R_{m}\cdot C_{m}\right)}{\sum_{m=1}^{12}C_{m}},\left(C_{m}\geq 5\right)$$

### 3.2. Classification of HSR Stations

To deeply understand the level of HSR station development and provide decision-making support for the operation and construction of new stations in the future, we classified the stations according to the brightness during operation and relative brightness changes compared to the time before the operation. A total of 708 stations began HSR operation in 2013–2019, and another 272 stations began HSR operation in 2003–2012. Since we could not obtain the brightness before the operation for stations that began operation before or in 2012, we developed classification standards based on the 708 stations that began operation in 2013–2019.

Suppose that a given station starts HSR operation in year *t*. First, the average brightness *R_before_* of each station of year (*t* − 2012) before the HSR operation (Formula (2)) and the average brightness *R_after_* of year (8 − (*t* − 2012)) during the HSR operation (Formula (3)) were calculated. Then, the relative brightness changes *C_rela_* after HSR operation opening (Formula (4)) was calculated. Since the median could exclude the influence of extremely large or small values and reflect the intermediate level of brightness more than the average, the median was selected as the threshold for dividing the station type.

First, the stations were divided into high-brightness or low-brightness categories according to the median *R_median_* of the average light *R_after_* of 708 stations during operation. Then, the stations were divided into large-change or small-change categories according to the median *C_median_* of the relative brightness changes *C_rela_* during operation compared to that before the operation. Combining the two classification thresholds, four categories were obtained: high brightness with large changes, high brightness with small changes, low brightness with large changes, and low brightness with small changes. For the 272 stations that started HSR operation from 2003 to 2012, the brightness before the operation was not available; hence, we only considered brightness during operation and used the same threshold *R_median_* as 708 stations to divide these stations into high-brightness or low-brightness categories.

(2) $$R_{before}=\frac{\sum_{y=2012}^{t-1}R_{y}}{t-2012}$$

(3) $$R_{after}=\frac{\sum_{y=t}^{2019}R_{y}}{2019-t+1}$$

(4) $$C_{rela}=\frac{R_{after}-R_{before}}{R_{before}}$$

### 3.3. Analysis of Light Brightness Influencing Factors

This research primarily considered the impact of two factors related to HSR operation on brightness. The impact of daily trains was realized by analyzing the number of trains of different station categories, and the results are presented through box plots and histograms. The impact of operation start time is realized by analyzing the annual brightness changes during operation and finding the years with the most considerable changes.

For the stations that start HSR operation in year *t*, the number of operation year *k* and annual brightness changes *C_k_* are shown in Table 1. Since the stations that started HSR operation before 2012 have been started for several years until 2019, the number of operation years is not within the expected study scope, and the annual change data are incomplete. Therefore, only the annual brightness changes of the 774 stations that started HSR operation in 2012–2019, which have 0 to (2019 − *t*) year of operation, were counted. We counted the average *C_k_mean_* of annual changes *C_k_* (0 ≤ *k* ≤ 7) in year *k* of all stations whose opening year is equal to or greater than *k* years. Then, for each station, the year *k*1 with the maximum annual changes was determined. Finally, we performed histogram statistics on *k*1 of each station and analyze its spatial distribution pattern.

## 4. Results

### 4.1. Spatial and Temporal Distribution of Light Brightness at HSR Stations

#### 4.1.1. Spatial Distribution

**Figure 3:**
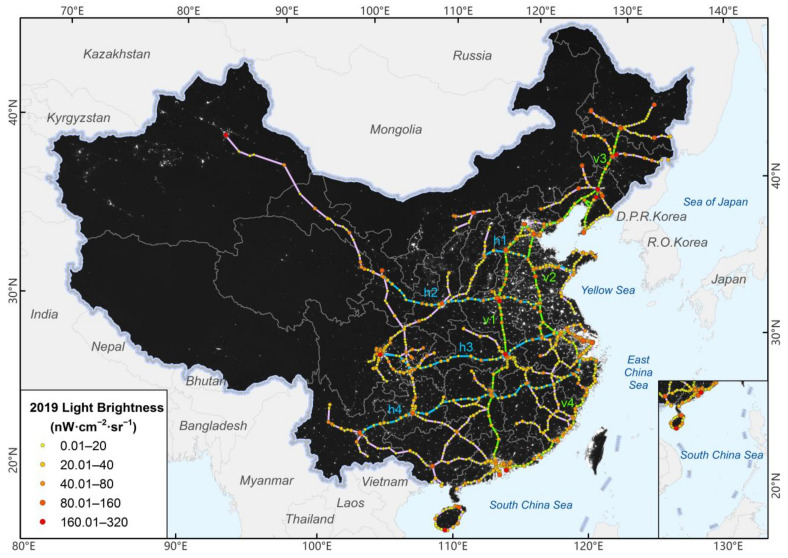
Spatial distribution of light brightness of HSR stations in 2019.

The maximum brightness within the range of 1.5 km × 1.5 km around 980 HSR stations in 2019 are shown in Figure 3. There are 41 stations with high light brightness between 80 and 320 nW·cm^−2^·sr^−1^ nationwide, which are mainly distributed in provincial capitals, large cities (e.g., Shenzhen, Suzhou, and Dalian), and the end of lines (e.g., Zhuhai, Tongliao, and Jiamusi). There are approximately 410 stations with a brightness between 20 and 80 nW·cm^−2^·sr^−1^ mainly concentrated in coastal areas, and 529 stations with brightness less than 20 nW·cm^−2^·sr^−1^ distributed widely in other areas.

Four vertical and four horizontal lines are the backbone of China’s HSR network, whose average brightness, maximum brightness, and number of stations are shown in Table 2. In general, the Beijing–Harbin–Dalian, Beijing–Guangzhou–Shenzhen, and Beijing–Shanghai lines have the highest average brightness, with an average brightness exceeding 35 nW·cm^−2^·sr^−1^. The brightness of the four vertical lines in the eastern coastal area was higher than that of the four horizontal lines connecting the eastern, central, and western parts. In the four vertical lines, the Beijing–Shanghai, Beijing–Guangzhou–Shenzhen, and Hangzhou–Shenzhen connected the Beijing–Tianjin–Hebei economic circle, the Yangtze River Delta economic circle, and the Pearl River Delta economic circle each other. The brightness of Shenzhen Futian Station on the Beijing–Guangzhou–Shenzhen line was 319.23 nW·cm^−2^·sr^−1^, ranking first among 980 stations in the country and more than twice that of Shanghai Hongqiao, because of the rapid development of Shenzhen after reform and opening up and the following large population gathering. Its high brightness was also related to its location in the central business district at Shenzhen’s core. In particular, due to the large-scale and high-density population in Henan, the lights in Anyang East, Hebi East, and Zhumadian West station in Henan province exceeded 50 nW·cm^−2^·sr^−1^, which were the highest, except for Shenzhen and provincial capitals on this HSR line. The average brightness of the Hangzhou–Shenzhen line was at minimum in four vertical lines with only 29.29 nW·cm^−2^·sr^−1^, and the maximum brightness was only 73.87 nW·cm^−2^·sr^−1^ at Puning station. Although it had 65 stations, much more than other major lines, it had the least stations over 60 nW·cm^−2^·sr^−1^, which was likely to be associated with the lower number of provincial capitals it passes through.

For the horizontal lines, the average brightness of Qingdao–Shijiazhuang–Taiyuan line, Xuzhou–Lanzhou line, and Shanghai–Wuhan–Chengdu line was approximately 30 nW·cm^−2^·sr^−1^. Among them, the Shanghai–Wuhan–Chengdu line had the largest number of stations over 60 nW·cm^−2^·sr^−1^ because of the quantity of important provincial cities it passed by. The Shanghai–Kunming line has recently been opened and passes through underdeveloped provinces such as Jiangxi, Hunan, Guizhou, and Yunnan; thus, the average brightness was relatively low at 20.4 nW·cm^−2^·sr^−1^, and there were only two stations over 60 nW·cm^−2^·sr^−1^ because the provincial cities it passed through were relatively underdeveloped. Hangzhou, Yiwu, Jinhua, and the ending point of Kunming had relatively high brightness, but other stations had brightness below 40 nW·cm^−2^·sr^−1^, including the provincial capitals of Nanchang, Changsha, and Guiyang.

#### 4.1.2. Temporal Distribution

**Figure 4:**
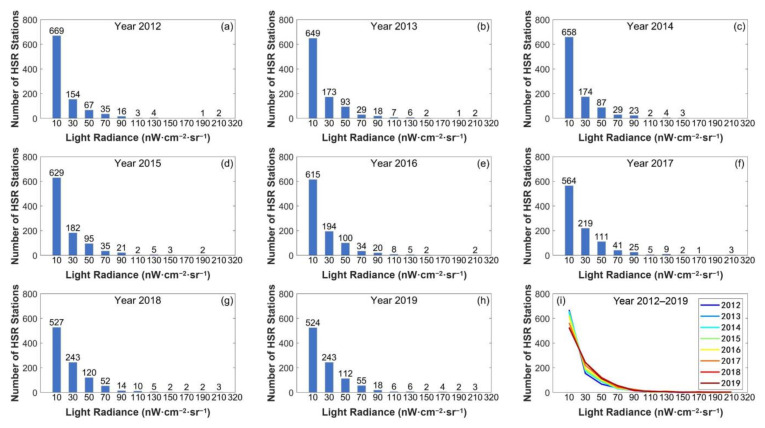
Light brightness of HSR stations from 2012 to 2019. (**a**–**h**) Frequency distribution histogram of brightness for each year from 2012 to 2019 and (**i**) variation curve of the number of stations with brightness from 2012 to 2019.

The brightness of 980 HSR stations from 2012 to 2019 is shown in Figure 4a–h. From 2012 to 2019, more than 75% of the stations were concentrated in the range of 0–40 nW·cm^−2^·sr^−1^, and more than half of the stations were below 20 nW·cm^−2^·sr^−1^. There were few stations with a brightness higher than 80 nW·cm^−2^·sr^−1^, and the distribution trend of brightness at HSR stations had not changed significantly. There is still great developing potential for China’s HSR system to bring deeper and broader impacts on human activity in the future. However, the number of low-brightness stations (i.e., brightness lower than 20 nW·cm^−2^·sr^−1^) decreased from 669 to 524, and the number of high-brightness stations (i.e., brightness greater than 60 nW·cm^−2^·sr^−1^) increased from 61 to 96, whose growth rate reaching 57%. In general, there is an increasing number of high-brightness stations, as shown in Figure 4i. The lights of 980 stations had moved to a high-brightness direction within eight years, indicating that the operation of HSR had an apparent driving effect on human activity and thus station development.

### 4.2. Classification of HSR Stations

#### 4.2.1. Distribution of Light Brightness and Relative Brightness Changes

**Figure 5:**
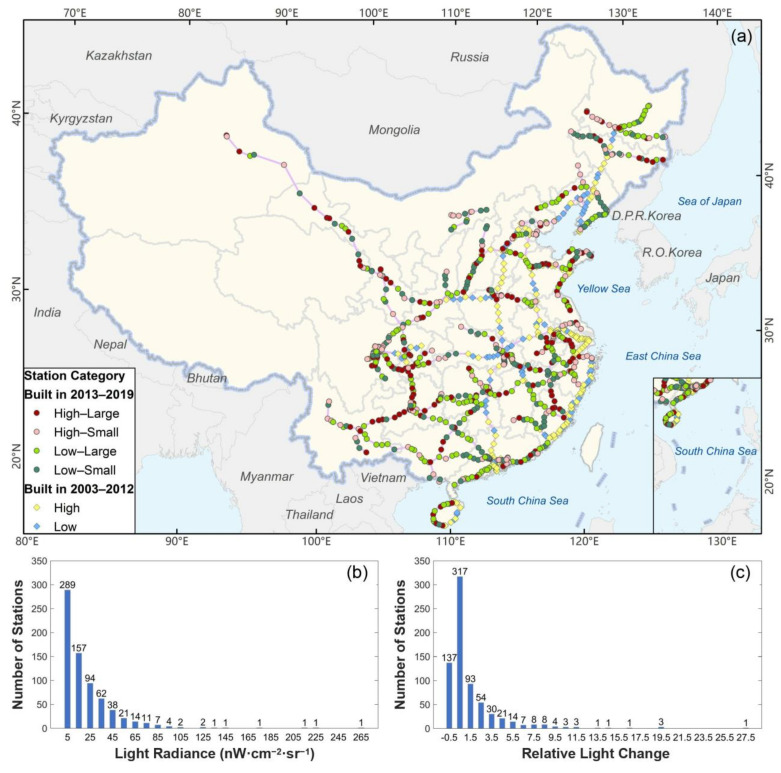
Classification basis and results of HSR stations. (**a**) Spatial distribution of HSR stations after classification. (**b**) Frequency distribution histogram of average brightness during operation for stations started HSR operation from 2013 to 2019 and (**c**) frequency distribution histogram of relative brightness changes for stations started HSR operation from 2013 to 2019.

The frequency distribution histogram of light radiance and relative brightness changes are shown in Figure 5b,c. More than 60% of the stations were concentrated in the range of 0–20 nW·cm^−2^·sr^−1^, and only ten stations had brightness greater than 100 nW·cm^−2^·sr^−1^, showing a long tail distribution. The relative changes of nearly half of the stations were within 100%, which means that the increase in nightlight did not exceed one time. The greater the amount of brightness changes, the fewer the number of stations. Only 75 stations increased the amount of light by more than four times. In particular, there were 137 stations with reduced light. Except for the brightness of some stations that fluctuate in a small range, the main reason is that the brightness during the construction period is higher than that during the operation period, such as Yangshuo in Guangxi, Huanggang in Hubei, Shexian North in Anhui, and Wendeng East in Shandong. These stations have quite a small passenger flow and thus low human activity intensity and light brightness. Some stations whose lights continued to decline during 2012–2019, such as Harbin North in Heilongjiang, Ningbo in Zhejiang, Lijiang in Yunnan, Weihai, and Yantai in Shandong. The cities where these stations are located are experiencing population loss and economic recession.

On the basis of a frequency distribution histogram, classifying HSR stations facilitates a deeper understanding of the different levels of station development and the nearby human activity for each station. The stations were classified according to the median of the average brightness during operation of the 708 stations opened in 2013–2019 *R_median_* and the median of the relative brightness changed compared to light before the operation *C_median_*. The median brightness of the 708 stations during HSR operation *R_median_* was 13.66 nW·cm^−2^·sr^−1^, and the median of relative brightness changes *C_median_* was 0.57.

#### 4.2.2. Station Categories

The stations that began HSR operation from 2013 to 2019 were divided into four categories: high-brightness and large-changes (high-large), high-brightness and small-changes (high-small), low-brightness and large-changes (low-large), and low-brightness and small-changes (low-small). There were 159 high-large and low-small stations each, and 194 high-small and low-large stations each, as shown in Figure 5a. High-large stations were mostly new HSR stations in provincial capitals or surrounding cities with high population density. There were many high-large stations in the Chengdu–Chongqing Circle, especially on Chengdu–Chongqing, Chengdu–Guiyang, and Chongqing–Guiyang lines. The formal formation of the Chengdu–Chongqing–Guiyang HSR ring will add another strong impetus to the economic growth in the west. Stations in Guizhou province on the Shanghai–Kunming line were mainly high-large stations, representing rapid and effective development of Shanghai–Kunming HSR operation in Guizhou province. High-small stations were mainly concentrated in old stations that operate both HSR and ordinary trains at the same time. There are multiple high-small stations around Guangzhou, Shenzhen, Chengdu, Wuhan, and Changsha. Stations in Zhejiang province, including those on Jinhua–Wenzhou, Hangzhou–Shenzhen, and Shanghai–Kunming lines, are high-small ones. The Harbin–Qiqihar line had the most high-small stations with only large changes on key nodes, including Anda and Qiqihar South stations. Low-large stations were concentrated in Harbin–Jiamusi, Harbin–Suifenhe, and Shenang–Chengde lines in three north-eastern provinces. There were also plenty of low-large stations in southern China on the Shenzhen–Zhanjiang, Kunming–Nanning–Guangzhou, and Hengyang–Huaihua lines. These towns remained undeveloped until HSR brought new opportunities for them through the flow of talents, technologies, and capital. There were stations with low brightness and small changes that did not gain much from HSR operation. A large number of stations on the Changchun–Baicheng–Ulanhot and Shenyang–Dandong–Dalian lines in the northeast belong to this category. In addition, the brightness of stations in Shanxi province on the Datong–Xi’an line did not grow much. Wuhan–Xiaogan, Wuhan–Xianning, and Wuhan–Huanggang–Jiujiang intercity railways in Hubei province have many low-small stations, similar to the Guiyang–Guangzhou, and Nanning–Guangzhou lines in Guangdong province.

The stations that started HSR operation from 2003 to 2012 were divided into two categories: high-brightness and low-brightness. Among them, there were 196 stations with high brightness and 76 stations with low brightness, as shown in Figure 5a. Early opened stations are generally located in cities with relatively developed economies and concentrated populations. Therefore, the brightness of these HSR stations was generally higher. The stations opened before or in 2012 were mainly concentrated on the Beijing–Harbin–Dalian line, Beijing–Guangzhou–Shenzhen line, Beijing–Shanghai line, Ningbo–Xiamen section of Hangzhou–Shenzhen line, Qingdao–Shijiazhuang–Taiyuan line, Zhengzhou–Xi’an section of Xuzhou–Lanzhou line, and Nanjing–Yichang section of Shanghai–Wuhan–Chengdu line. The low-brightness stations opened in 2012 and before mainly concentrated on the Shenyang–Shanhaiguan, and Shenyang–Dalian sections of Beijing–Harbin–Dalian line, the territory of the Hunan province of the Beijing–Guangzhou line, the central Zhejiang province of Hangzhou–Shenzhen line, Hubei province of Shanghai–Wuhan–Chengdu line, and stations in the central part of Nanchang–Jiujiang Intercity Railway.

### 4.3. Analysis of Influencing Factors

#### 4.3.1. Daily Trains

**Figure 6:**
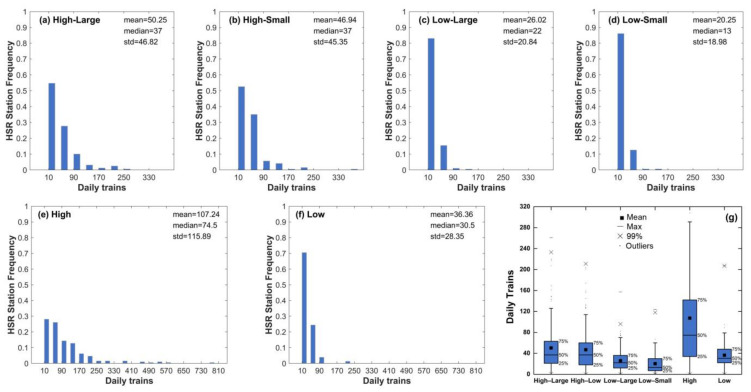
Impact of HSR daily trains on brightness. (**a**–**f**) Frequency distribution histogram of daily trains for different type of stations and (**g**) box chart of daily trains for different station types.

As shown in Figure 6g, the median and average of daily trains in the high-value category were larger than those in the low-value category, which demonstrated a positive correlation between daily trains and brightness to some extent. Stations with more daily trains have larger passenger flow; thus, they are more likely to have high brightness as a representative of the station development and human activity intensity. The median and average of daily trains in the large-change category are larger than those in the small-change category. Stations with more trains have more significant changes in brightness after the operation of HSR, indicating that the opening of HSR has a positive impact on station development and human activity intensity. Generally speaking, the more trains, the greater the degree of impact. High-brightness stations that started operation before or in 2012 had the largest median and average value in daily trains. On the one hand, there were more high-speed trains in economically developed and densely populated areas. On the other hand, due to the increasing number of high-speed trains, the flow of technology, talents, and capital can be accelerated, and the level of economic development and human activity intensity in the region will also improve. The level of human activity intensity and HSR daily trains complement each other and promote each other. This study used brightness to characterize the level of station development and nearby human activity intensity, and therefore there is an apparent positive correlation between brightness and daily trains. Stations that started HSR operation before 2012 were most located in developed areas with large populations; thus, they had the highest median and average values in daily trains.

The histogram of the daily train frequency distribution of different types of the station is shown in Figure 6a–f. The number of daily trains of high-brightness stations was more widely distributed in 41–200, especially for stations that started HSR operation in 2012 and before. On the contrary, the number of daily trains of low-brightness stations was mostly concentrated in the range of 1–40. In general, stations with more daily trains were more likely to have high brightness.

#### 4.3.2. Operation Start Time

**Figure 7:**
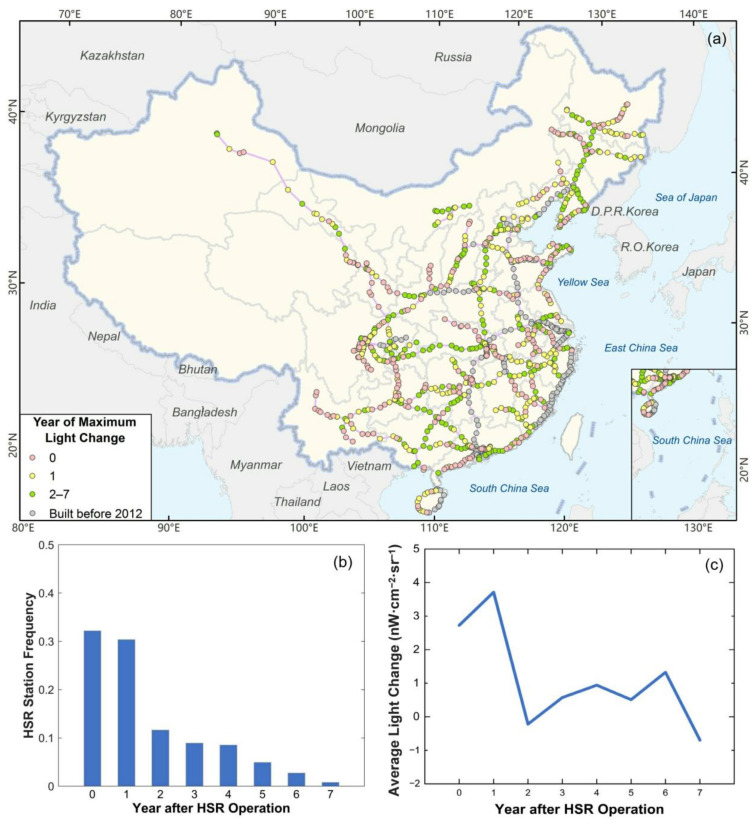
Impact of HSR operation start time on brightness. (**a**) Spatial distribution of years with the most considerable brightness changes during HSR operation; (**b**) frequency distribution histogram of years with the most considerable brightness changes; and (**c**) average brightness changes in the *k*th year of all stations that have started HSR operation for *k* years or more.

The average *C_k_mean_* of annual brightness changes *C_k_* (0 ≤ *k* ≤ 7) for the 774 stations, which started HSR operation in 2012 or after are shown in Figure 7c. Within 0–1 year, after HSR operation begins (0 represents the year when HSR starts operation), the annual change in brightness is the largest. The average brightness changes in the operation starting year were 2.73 nW·cm^−2^·sr^−1^, and those in the first year during operation reached 3.71 nW·cm^−2^·sr^−1^. Afterward, the overall rate of change declined, but the brightness increased steadily within 2–6 years. The average change in the second year during operation was –0.21 nW·cm^−2^·sr^−1^, but it increased to 1.33 nW·cm^−2^·sr^−1^ in the 6th year during operation. The results show that HSR had the most significant impact on station development and human activity intensity in the first year during operation, and then had a relatively long-term but slow impact in 2–6 years.

The years during operation corresponding to the maximum brightness changes are shown in Figure 7b. The maximum brightness changes were mostly distributed within 0–1 year after HSR operation opening, whose stations’ frequency exceeded 0.6. This proved that the impact of HSR on station development and nearby human activity intensity was most evident within 0–1 year after operation begins.

Furthermore, the spatial distribution of years with the most significant brightness changes after HSR operation opening for each station is shown in Figure 7a. Excluding newly opened stations in 2018 and 2019, the Changchun–Baicheng–Wulanhaote, Nanjing–Anqing, Nanchang–Fuzhou–Yongtai–Putian lines, and Hainan roundabout passenger dedicated lines had the most considerable annual changes in the year of opening or one year after opening. Beijing–Harbin, Harbin–Qiqihar, Shenyang–Dandong, Huhehaote–Baotou line, Wuhan–Chengdu section of Shanghai–Wuhan–Chengdu line, Hengyang–Liuzhou–Nanning, Guiyang–Guangzhou Passenger Dedicated line generally exhibit lag, and the most considerable amount of brightness changes appeared in 2–7 years after operation began.

## 5. Discussion

With the rapid development of HSR in China, evaluating HSR station development and nearby human activity and exploring its influencing factors, such as daily trains and operation start time, will help to widely and deeply understand HSR stations’ status in operation. Therefore, this study used brightness as a representative of station development and nearby human activity, analyzing its spatial and temporal distribution, classification categories, and influencing factors, which can provide valuable decision-making support for the government and other relevant departments to plan future HSR lines and choose the location of HSR stations. Furthermore, it contributes to more efficient HSR operation and reasonable and complete facilities nearby. It also helps for better allocating financial resources for public investment [52].

In this study, we found that stations with relatively higher brightness ranging in 80–320 nW·cm^−2^·sr^−1^ are basically concentrated in provincial capitals, large cities, and the end of lines and the number increased by 57% in the past 8 years, which demonstrated that light brightness could provide a fair assessment of the impact degree of China’s HSR on station development and nearby human activity intensity. We used the maximum brightness in the area of 1.5 km × 1.5 km around the stations to represent the station development and human activity intensity. According to Schütz [53], the areas outside the stations can be classified into primary, secondary, and tertiary areas, corresponding to 5–10 min, within 15 min, and more than 15 min on foot or seamless transport away from stations. The impacts are greatest in primary areas, whose land type generally consists of high-end office buildings and residential areas and can bring about growth in land prices and development in property [54,55]; thus, a circular area is often used with a 10-min walking distance of 500 m as the primary impact area, which is consistent with the 1.5 km × 1.5 km area given the area occupied by the station itself. Actually, HSR stations have a wider impact on the surrounding, and the hinterland of HSR can be larger in the process of improving economic activities and promoting urban expansion. Some literature discussed the scope of the spatial spillover effects. Huang found that HSR operation has an agglomeration shadow effect on urban economic activities, with positive effects within 80 min driving distance and inhibitory effects within 80–260 driving distance in China [43]. Ahlfeldt found it is reduced by 50% every 30 min and reduced to 1% after 200 min [13]. Pan found that HSR has a significant impact on areas within 5 km [56]. In future research, in addition to the primary areas that are most affected by HSR operation, we could also try to study the spatial extent of the impact of the HSR stations on the surrounding areas.

As the necessity of existing nodes to be classified pointed out by Priemus [57], the classification of 980 stations helps to analyze in-depth the balance of development brought about by HSR. We found that new stations in the suburbs were more likely to have large brightness changes, and they were often located in provincial capitals or surrounding cities. The old stations in the urban area of large cities and stations in cities far from provincial capitals gain less from HSR operation. Our results show heterogeneity of the effects brought by HSR according to the distance between cities and provincial capitals and the distance between stations and city centers, which represents inequity. On the one hand, HSR should maximize the benefits according to the cost–benefit analyses [58], which means serve large cities with a huge population as much as possible. On the other hand, as a public infrastructure invested by the governments, it should also take intermediate and small cities and towns into consideration for the balance of development [59]. Consequently, the development of HSR infrastructure requires both efficiency and equity, and thus it aroused widespread attention to the differences in the HSR impacts and the main factors in the academic world. Ke found that cities with positive effects are mainly located in transportation hubs in China’s coastal areas [60]. Chen found HSR has positive impacts on stations within a distance of 2 h from London [61]. Stations close to large cities can accept industrial spillovers such as Dongguan and Foshan in Guangdong Province, while those far away from developed cities are more likely to have a net outflow of labor. Yin found that large cities with international service capabilities could attract more talents and financial investment to enhance the economic agglomeration effects due to the improved accessibility [62]. Moyano studied about four mediate and small cities in Europe and found drawbacks, including local markets that are too small for long-distance travel and inappropriate station design [24]. The above two studies show the economy, industry, and population also plays a crucial role in HSR impacts. In general, feedback on the HSR operation is heterogeneous with more benefits for stations in cities with higher economic potentials on appropriate locations. The factors include the ability of investment and labor absorbing, better infrastructure and even tourist attractions.

Literature shows that HSR operation is a much more critical factor considering urban development compared to HSR infrastructure, including lines and stations [24]. Consequently, we took daily trains into account as an important factor related to HSR operation. The factor of daily trains directly represents the demand for transportation of the city, which is an indicator of both population and economic activities and the light brightness. Thus the daily trains and brightness show an interdependent model in the positive correlation, in which they represent each other and promote each other. We may focus on more HSR operation parameters, including routes, timetable [24], cost, and passengers using the service [52] in the future study. As for HSR operation start time, Pan analyzed the relationship between the impact of HSR on the surroundings and operation start time on a more detailed monthly scale, and it was found that the spatial spillover effect generally lags 3–6 months [56]. This is consistent with the conclusion we got on the annual scale that the brightness changes fastest in 0–1 year after the operation.

There is a condition in which HSR development and urban development reinforce each other. For example, after the completion of the subway in London and Paris, the accessibility of the city center has been improved, and the connection between the city center and the suburbs has been strengthened, which has greatly promoted the development of the city. The development, in turn, determines the operation of the existing transportation system and the future planning of the transportation system [62]. Studies have shown that this phenomenon exists in different types of cities and different transportation systems [63]. In the node-place model, improving the transportation capacity of the node can increase the intensity and diversity of surrounding land use, and in turn, increasing the value of the surrounding land can create traffic demand [62]. The developed cities with quantity of economic activities and large consumer markets first attract investment in HSR and justify the high-frequency services, then the agglomeration of capital and talents is strengthened due to the strong economic foundation, favorable policy orientation, advanced technology, and completed infrastructure. The medium-sized or small cities with advantaged geographical location and unique resource endowment are selected to pass through and gain economic growth with industry spillover and the rise of characteristic industries.

In summary, this study provided suggestions for the government of different level of cities. First of all, for intermediate and small cities with HSR operations but have no absolute advantage in geographical location, investment in hardware (e.g., public transportation infrastructure, supporting facilities, and industrial parks) and software (e.g., industry introduction subsidies) should be strengthened. In particular, the integrated traffic capacity around HSR stations should be given the most attention, including connections with subways, buses, taxis, and parking spaces. Secondly, the central government should build new HSR lines and stations in cities or towns with resource endowment, large population, industrial foundation, and basic infrastructure. HSR will bring a positive spillover effect to the local area only if these conditions are met, introducing more external capital without causing labor loss. Thirdly, regarding HSR stations’ location in new cities or towns, the government should implement a multicenter space strategy and build HSR stations within 80 minutes’ drive from the city center.

In future research, improvements can be made from the following three aspects. The first is to combine high-precision construction land data to explore the scope of station impact on the surrounding area in various cities and locations. The second is to combine DMSP/OLS data to conduct longer-term analyses starting from 2008 when the first HSR line began operation in China. The third is to consider the relationship between light brightness with external conditions and the stations’ operation situations. The external conditions include social (e.g., population, employment, and tourism), economic (e.g., GDP and industrial structure), and policy factors; while operation situations include the distance of stations from central building districts (CBDs), capacity of the integrated transportation system, and the level of infrastructure.

## 6. Conclusions

This study used NPP/VIIRS nighttime light data to evaluate HSR station development and nearby human activity intensity. First, the spatial and temporal distribution of brightness of 980 HSR stations nationwide in 2012–2019 was evaluated. Then, the HSR stations were classified into four categories according to the brightness during operation and brightness changes compared to that before the operation. Finally, the impact of daily trains and operation start time on brightness was explored to understand how HSR operation affected station development and human activity intensity. We mainly drew the following three conclusions:

(1) There were 41 stations with high brightness between 80 and 320 nW·cm^−2^·sr^−1^, which were basically concentrated in the provincial capitals, large cities, and the end of lines. Among the four vertical and four horizontal major HSR lines, Beijing–Harbin–Dalian, Beijing–Guangzhou–Shenzhen, and Beijing–Shanghai lines had the highest average brightness exceeding 35 nW·cm^−2^·sr^−1^, while the Shanghai–Kunming line had the lowest average light of only 20.40 nW·cm^−2^·sr^−1^. In the time series from 2012 to 2019, more than 75% of the station lights were concentrated below 40 nW·cm^−2^·sr^−1^; but in general, the station lights moved to the high-brightness direction in the past eight years with 57% more high-brightness stations and 145 fewer low-brightness stations.
(2) Among the stations that began HSR operation in 2013–2019, 159 were high-brightness stations with considerable changes, 194 were high-brightness stations with small changes, 194 were low-brightness stations with considerable changes, and 159 were low-brightness stations with small changes. In general, stations with high brightness and large changes were mainly new HSR stations in provincial capitals or surrounding cities. There were numerous high-large stations on the Chengdu-Chongqing-Guiyang HSR ring and in Guizhou Province on the Shanghai-Kunming line. High-brightness stations with small changes were mainly old stations operating both HSR and ordinary trains. More than 70% of stations that began HSR operation in 2003–2012 belonged to high-brightness categories due to their locations in cities with relatively developed economies and concentrated populations.
(3) Daily trains and HSR operation start time are two primary factors that have a close relationship with brightness. Daily trains of high-brightness stations are more than those of low-brightness stations, and HSR stations in the large-change category have more daily trains than the small-change category, which proves that brightness has a positive correlation with daily trains and lights increased faster when more daily trains bring large passenger flow. As daily trains and brightness are both indicators of population and level of economic development, they are interdependent, representing, and complementing each other. From the perspective of HSR operation start time, the lights increased most in the starting and following year of HSR operation. Maximum brightness changes appeared within 0–1 year during operation for more than half of the evaluated stations. Then, 2–6 years after operation, HSR exhibited a relatively slow but long-term impact on station development and nearby human activity intensity.

## Figures and Tables

**Table 1:** Years of operation and annual brightness changes for stations starting HSR operation in year *t*.

Year	*t*	*t* + 1	……	2019
Years of operation *k*	0	1	……	2019 − *t*
Annual brightness changes *C_k_*	/	*R*_*t*+1_ − *R_t_*	……	*R_2019_*_−*t*_ − *R_2018_*_−*t*_

**Table 2:** Average brightness of four vertical and four horizontal HSR lines in 2019.

Line Code	Line Name	Average Brightness	Maximum Brightness	Station with Maximum Brightness	Number of Stations	Number of Stations over 60 nW·cm^−2^·sr^−1^
v1	Beijing –Guangzhou –Shenzhen	36.72	319.23	Futian	44	6
v2	Beijing –Shanghai	42.01	146.83	Shanghai Hongqiao	43	11
v3	Beijing –Harbin –Dalian	44.50	172.57	Liaoyang	46	14
v4	Hangzhou –Shenzhen	29.29	73.87	Puning	65	5
h1	Qingdao –Shijiazhuang –Taiyuan	32.51	77.07	Jinan East	24	3
h2	Xuzhou –Lanzhou	29.95	130.49	Xi’an North	33	3
h3	Shanghai –Wuhan –Chengdu	30.17	85.49	Hankou	34	5
h4	Shanghai –Kunming	20.40	78.74	Kunming South	46	2

## Data Availability

Publicly available datasets were analyzed in this study. This data can be found here: [https://developers.google.com/earth-engine/datasets/catalog/NOAA_VIIRS_DNB_MONTHLY_V1_VCMCFG#description].

## Supplementary Materials

The following are available online at https://www.mdpi.com/1660-4601/18/2/557/s1, Figure S1: Cloud-free coverage of HSR stations from 2012–2019, Figure S2: Cloud-free observation histogram corresponding to the maximum brightness. Reference [64] is cited in the supplementary materials.
