# Supplementary material for: Evaluation of China’s High-Speed Rail Station Development and Nearby Human Activity Based on Nighttime Light Images
Source: Int J Environ Res Public Health. 2021 Jan 11;18(2):557. doi: 10.3390/ijerph18020557 (PMC7827851; doi:10.3390/ijerph18020557)
Supplement: Supplementary file 1 [file ijerph-18-00557-s001.pdf]

The number of cloud-free observations of all data in the study area pixel-by-pixel is shown in Supplement Figure 1. The nighttime light in the northern area in high latitudes is polluted by stray light in summer [1], which causes a certain degree of data missing from May to August of some stations. However, due to the large quantity of sunny days in northern areas, there is still a large amount of cloud-free observations in the 8-year time series. Southern regions such as the Yangtze River Delta and the Pearl River Delta have cloudier and rainy days; thus, the cumulative value of cloud-free observations is relatively small. Sichuan and Guizhou province are almost cloudy throughout the year and there are few cloud-free observations; thus, the data quality is affected to a relatively large extent. In general, the average cloud-free observation value in 8 years of 980 HSR stations reached 662, which is of high credibility.

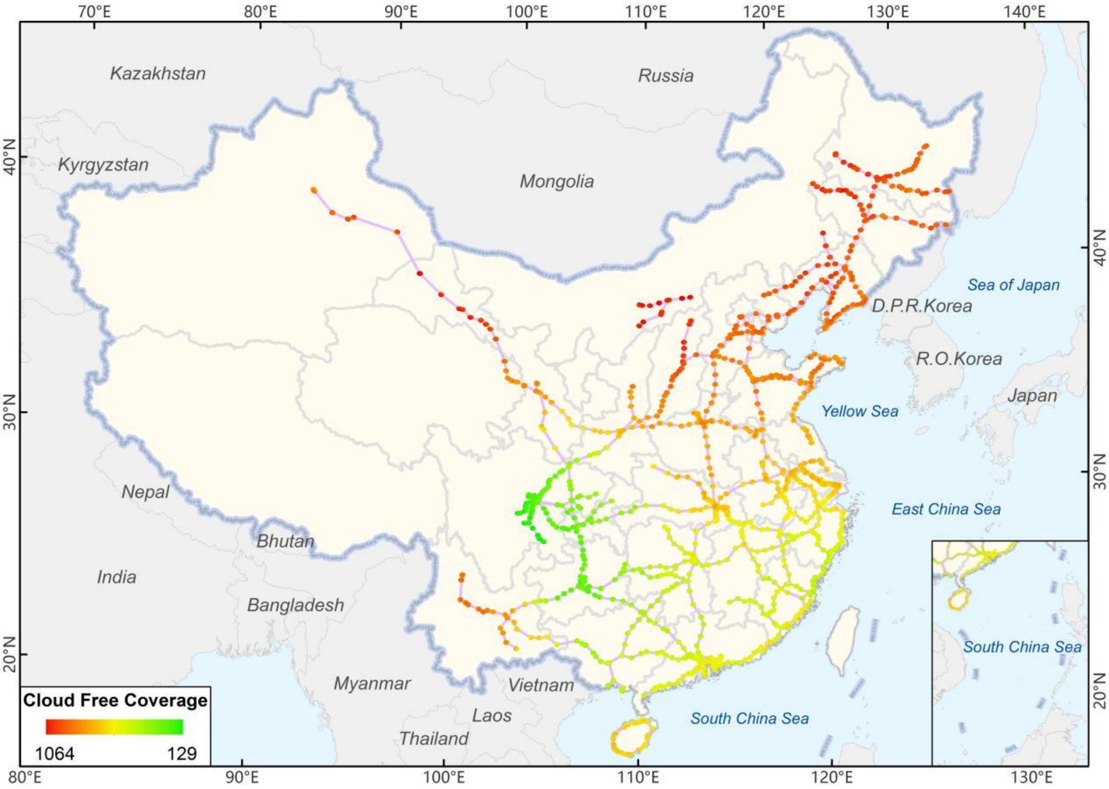


**Figure S1.** Cloud-free coverage of HSR stations from 2012 to 2019

The number of cloud-free observations where the maximum brightness was located at 980 stations in 89 months is shown in Supplementary Figure 2. The range of cloud-free observation of a single scene of an image is within 0–25. There are 6419 pixels without cloud-free observations affected by clouds or stray light. The number of pixels initially increased and then decreased as cloud-free coverage increased from 1 to 25.

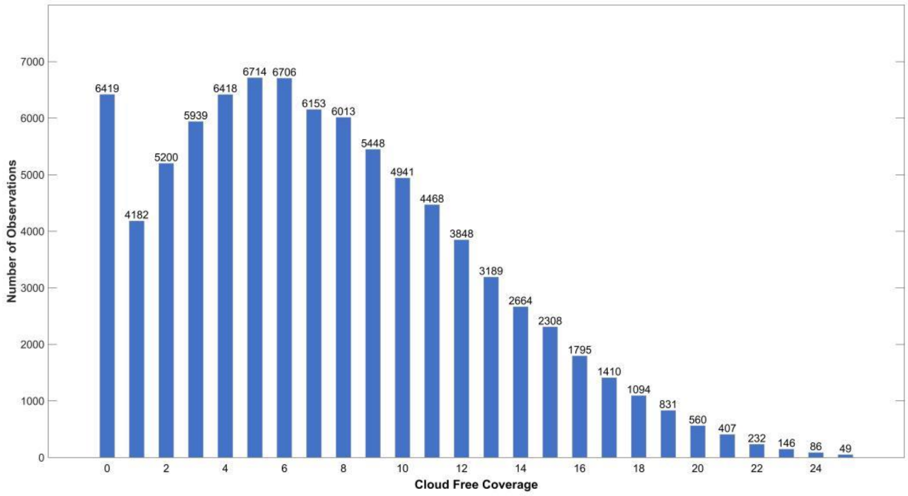


**Figure S2.** Cloud-free observation histogram corresponding to the maximum brightness

## References


1. Chen, M.; Cai, H., Interpolation methods comparison of VIIRS/DNB nighttime light monthly composites: A case study of Beijing. *Prog. Geography* **2019**, *38*, 126–138.
